# The association of very-low-density lipoprotein with ankle-brachial index in peritoneal dialysis patients with controlled serum low-density lipoprotein cholesterol level

**DOI:** 10.1186/1471-2369-14-212

**Published:** 2013-10-07

**Authors:** Eiichiro Kanda, Masumi Ai, Mitsuyo Okazaki, Yoshitaka Maeda, Sei Sasaki, Masayuki Yoshida

**Affiliations:** 1 Department of Nephrology, Tokyo Kyosai Hospital, Nakameguro 2-3-8, Meguroku, Tokyo 153-8934, Japan; 2 Bioethics Research Center, Tokyo Medical and Dental University, Yushima 1-5-45, Bunkyoku, Tokyo 113-8519, Japan; 3 Department of Nephrology, Tokyo Medical and Dental University, Yushima 1-5-45, Bunkyoku, Tokyo 113-8519, Japan; 4 Skylight Biotech Inc, 100-4 Sunada Iijima-aza, Akita 011-0911, Japan; 5 Department of Nephrology, JA Toride Medical Center, Hongo 2-1-1, Toride Ibaraki, Japan

**Keywords:** Peritoneal dialysis, Ankle-brachial index, Very low density lipoprotein, Atherosclerosis, Peripheral artery disease, Chromatography

## Abstract

**Background:**

Peripheral artery disease (PAD) represents atherosclerotic disease and is a risk factor for death in peritoneal dialysis (PD) patients, who tend to show an atherogenic lipid profile. In this study, we investigated the relationship between lipid profile and ankle-brachial index (ABI) as an index of atherosclerosis in PD patients with controlled serum low-density lipoprotein (LDL) cholesterol level.

**Methods:**

Thirty-five PD patients, whose serum LDL cholesterol level was controlled at less than 120mg/dl, were enrolled in this cross-sectional study in Japan. The proportions of cholesterol level to total cholesterol level (cholesterol proportion) in 20 lipoprotein fractions and the mean size of lipoprotein particles were measured using an improved method, namely, high-performance gel permeation chromatography. Multivariate linear regression analysis was adjusted for diabetes mellitus and cardiovascular and/or cerebrovascular diseases.

**Results:**

The mean (standard deviation) age was 61.6 (10.5) years; PD vintage, 38.5 (28.1) months; ABI, 1.07 (0.22). A low ABI (0.9 or lower) was observed in 7 patients (low-ABI group). The low-ABI group showed significantly higher cholesterol proportions in the chylomicron fraction and large very-low-density lipoproteins (VLDLs) (Fractions 3–5) than the high-ABI group (ABI>0.9). Adjusted multivariate linear regression analysis showed that ABI was negatively associated with serum VLDL cholesterol level (parameter estimate=-0.00566, *p*=0.0074); the cholesterol proportions in large VLDLs (Fraction 4, parameter estimate=-3.82, *p*=0.038; Fraction 5, parameter estimate=-3.62, *p*=0.0039) and medium VLDL (Fraction 6, parameter estimate=-3.25, *p*=0.014); and the size of VLDL particles (parameter estimate=-0.0352, *p*=0.032).

**Conclusions:**

This study showed that the characteristics of VLDL particles were associated with ABI among PD patients. Lowering serum VLDL level may be an effective therapy against atherosclerosis in PD patients after the control of serum LDL cholesterol level.

## Background

Peripheral artery disease (PAD) is frequently observed among patients with end-stage renal disease (ESRD) [1]. A cross-sectional study showed that the prevalence of PAD was 27.4% in 343 PD patients [2]. In accordance with the Trans-Atlantic Inter-Society Consensus 2 for the management of PAD (TASC2), PAD is diagnosed using the ankle-brachial index (ABI) [3].

PAD is associated with increased mortality and cardiac mortality among hemodialysis patients [4,5]. A single-center cohort study of 153 PD patients showed that a decrease in ABI increased the risk of death [6]. PAD represents atherosclerotic disease. In the Reduction of Atherothrombosis for Continued Health registry, PAD was observed with polyvascular disease [7]. The risk factors for PAD are diabetes mellitus (DM), age, and serum albumin, triglyceride (TG) and serum cholesterol levels [2,6,8]. Although PAD is prevalent in PD patients, it remains to be clarified which factors affect the progression of atherosclerosis and the development of PAD in PD patients.

Dyslipidemia is considered as one of the risk factors for PAD in TASC2 [3]. PD patients tend to have atherogenic lipid profiles, such as high levels of total cholesterol, LDL cholesterol (LDL-C), TG, and small dense LDL-C with a low level of high-density lipoprotein cholesterol (HDL-C) [9]. The kidney disease outcomes quality initiative (K/DOQI) showed that 78.6% of PD patients need treatment for dyslipidemia [10]. The Japanese Society of Nephrology and the Japanese Society for Dialysis Therapy guidelines recommend that serum LDL-C level should be maintained at less than 120 mg/dl [11,12]. However, the effect of lipid profiles on atherosclerosis has not been clarified in PD patients with controlled serum LDL-C levels.

We previously established a method, namely, high-performance gel permeation chromatography (HPGPC) that can separate lipoproteins into 20 fractions on the basis of differences in particle size [13,14]. The HPGPC method measures all lipoprotein subclasses in a single analysis from a very small amount of whole serum or plasma in a very short time. We successfully applied this technique to show that the visceral fat area is positively correlated with cholesterol levels in very-low-density lipoprotein (VLDL) and low-density lipoprotein (LDL) subclasses, but negatively with those in large and high-density lipoprotein (HDL) subclasses in male subjects with visceral fat syndrome or obesity, and that cardiovascular disease patients had a significantly higher cholesterol levels in small VLDL, small LDL, and very small LDL subclasses, but a lower cholesterol level in large HDL [13], [15].

In this study, we investigate the effects of lipid profile at subclass levels and the size of lipoproteins on ABI as a marker of atherosclerosis in the peripheral artery in PD patients with serum LDL-C level controlled at less than 120 mg/dl by HPGPC.

## Methods

### Study design and study population

This is a cross-sectional study of PD patients (more than 20 years of age) treated at Tokyo Kyosai Hospital, Tokyo, Japan, and JA Toride Medical Center, Toride, Japan, from October 2011 to December 2011, which was approved by the local human research ethics committee of Tokyo Kyosai Hospital, and JA Toride Medical Center. Written informed consent was obtained from each patient. Patients were eligible for inclusion in this study if they were able to stably undergo continuous ambulatory PD or automated PD as outpatients. They were treated with dialysates containing dextrose (Dianeal-NPD-2, -NPD-4 1.5 or 2.5, Baxter Japan, Tokyo; Mid Peric or Mid Peric L 135 or 250, Terumo, Tokyo) and a dialysate with or without icodextrin (Extraneal, Baxter Japan, Tokyo). We adhered to the Japanese Society for Dialysis Therapy guidelines for treatment by PD [16]. Dyslipidemia was diagnosed on the basis of the criteria of the Japan Atherosclerosis Society [17]. A high LDL-C level was treated in accordance with the evidence-based practice guideline 2009 for the treatment of chronic kidney disease of the Japanese Society of Nephrology and clinical guidelines for the evaluation and treatment of cardiovascular complications in hemodialysis patients of the Japanese Society for Dialysis Therapy [11,12]. Serum LDL-C level was maintained at less than 120mg/dl by administration of statin. Fibrates, ezetimibe, probucol, resins, other lipid lowering drugs and antioxidant drugs were not used in the patients of this study. We excluded patients who had malignant diseases, infectious diseases, or severe liver diseases.

### Data

Patient demographics including age; gender; comorbid conditions such as DM, hypertension, and dyslipidemia; statin use; and history of cardiovascular and/or cerebrovascular diseases (CVDs) were obtained from the medical records of the patients at each hospital. None of the patients were administered ezetimibe or fibrates. ABI was measured using an analyzer, Vascular Screening System VaSera VS-1500N (Fukuda Denshi Co., Tokyo, Japan). ABI was calculated using the ratio of systolic blood pressure in the ankles to systolic blood pressure in the arms, which was derived by taking the mean of the right and left ratios. We defined ABI to be low on the basis of the criteria of TASC2 [3]. The patients who had a low ABI (0.9 or lower) were classified into the low-ABI group and others into the high-ABI group. Blood samples were obtained from each patient after a 12 hour overnight fasting with an overnight dwell of dialysate containing 2.5% dextrose. Serum samples were dispensed into three tubes, one for routine serum biochemistry at each hospital, one for measurement of apolipoprotein B at SRL Inc., Tokyo, Japan, and one for HPGPC at Skylight Biotech Inc., Akita, Japan. The blood samples for HPGPC were frozen at -80°C immediately after their collection until use. Routine serum biochemistry was carried out by standard methods at each hospital. Albumin-corrected serum calcium level was calculated as follows: albumin-corrected serum calcium level (mg/dL) = measured serum calcium level (mg/dL) + (4.0 - serum albumin level [g/dL]), when the serum albumin level was less than 4.0 g/dL. Intact parathyroid hormone (PTH) level was measured using an immunoassay system (ARCHITECT Intact PTH, Abbott Japan Co., Chiba, Japan). Non-HDL-C level was calculated as the difference between total cholesterol and HDL-C levels. Apolipoprotein B was measured using an immunoassay kit (Apolipoprotein B; Sekisui Medical Co., Tokyo, Japan). Lipoprotein fractions were analyzed by HPGPC as previously described [13,14]. In brief, 4 μL aliquots of whole serum samples were injected into columns and what was continuously monitored at 550 nm after an online enzymatic reaction. Cholesterol level and the proportion of cholesterol level in each lipoprotein fraction to total cholesterol level (cholesterol proportion) were calculated mathematically with modified Gaussian curve fitting to resolve overlapping peaks: chylomicrons (CMs) [Fraction 1 (F1) and F2], large VLDL (F3-F5), medium VLDL (F6), small VLDL (F7), LDL (F8), medium LDL (F9), small LDL (F10), very small LDL (F11-F13), very large HDL (F14-F15), large HDL (F16), medium HDL (F17), small HDL (F18), and very small HDL (F19-F20) [13,14]. Considering the diameters of LDL particles, medium, small, and very small LDLs (F9 - F13) are consistent with the classification of small dense LDL particles by Okazaki et al. and Austin et al. [13,18]. The cholesterol and TG proportions in F1 and F2 were combined and treated as those in the CM fraction. The mean sizes of very-low-density lipoprotein cholesterol (VLDL-C), LDL-C, and HDL-C particles were measured by HPGPC using a calculation based on the defined size of lipoprotein particles and the cholesterol proportion in lipoprotein fractions [13,19].

### Statistical analyses

Normally distributed variables are presented as mean and standard deviation; otherwise, the median and interquartile range are presented. For parameters not normally distributed, intergroup comparisons were performed using the chi-square test, t test, and Mann–Whitney U test as appropriate. For parameters not normally distributed, natural logarithm values were considered in tests that require normally distributed variables after the tests of their normality: the natural logarithm values of CM cholesterol (CM-C) level [log(CM)] and TG level [log(TG)]. Univariate linear regression analysis was carried out to determine the relationships of ABI with the clinical and biochemical characteristics of patients. Multivariate linear regression analysis, which was adjusted for factors that were previously selected among the patient characteristics, were carried out to identify the factors that were independently associated with ABI. The relationships between the size of lipoprotein particles and serum cholesterol and TG levels or the cholesterol proportion in lipoprotein fractions were evaluated using Pearson’s correlation coefficients or Spearman’s rank correlation coefficient as appropriate. These analyses were conducted using SAS, version 9.2 (SAS, Inc., North Carolina, US). Statistical significance was defined as *p*<0.05.

## Results

### Patient characteristics

Thirty-five patients on PD were included as subjects for analysis. Of these, 28 showed high ABI levels (high-ABI group) and seven patients showed low ABI levels (low-ABI group). The patient demographics including biochemical data are shown in Table 1. Only one patient had an ABI (1.61) higher than 1.4. The prevalences of DM and history of CVD were higher in the low-ABI group, and the serum glucose levels of the low-ABI group were higher than those of the high-ABI group. Hemoglobin A1c were measured among DM patients; low-ABI group, 6.3±0.9%; high-ABI group, 6.2±0.6%. The distributions of cholesterol and TG levels in lipoprotein classes were examined between the high- and low-ABI groups (Table 2). The VLDL-C and TG levels in the low-ABI group were higher than those in the high-ABI group.

**Table 1:** Clinical and biochemical characteristics of patients

	**All**	**High-ABI group**	**Low-ABI group**	***p***
N (%)	35	28	7	
Male (%)	28 (80)	22 (78.6)	6 (85.7)	0.66
Age	61.6 ± 10.5	60.8 ± 10.7	64.9 ± 9.9	0.36
Height (cm)	163.0 ± 9.6	163.8 ± 9.9	159.8 ± 8.5	0.33
Weight (kg)	60.8 ± 11.1	61.6 ± 12.1	57.4 ± 4.6	0.38
BMI	22.8 ± 3.4	22.9 ± 3.5	22.7 ± 3.3	0.91
DM (%)	15 (42.9)	9 (32.1)	6 (85.7)	0.009*
Hypertension (%)	31 (88.6)	26 (92.9)	5 (71.4)	0.15
Dyslipidemia (%)	17 (48.6)	14 (50.0)	3 (42.9)	0.74
Statin use (%)	12 (34.3)	9 (32.1)	3 (42.9)	0.60
CVD (%)	9 (25.7)	4 (14.3)	5 (71.4)	0.003*
Icodextrin use (%)	18 (51.4)	14 (50.0)	4 (57.1)	0.74
Creatinine (mg/dl)	10.4 ± 3.1	10.4 ± 3.4	10.4 ± 1.4	0.97
Albumin (g/dl)	3.3 ± 0.4	3.3 ± 0.4	3.1 ± 0.4	0.40
Glucose (mg/dl)	114.7 ± 32.9	108.9 ± 24.4	141.5 ± 53.7	0.025*
Albumin-corrected serum calcium (mg/dl)	9.8 ± 0.7	9.7 ± 0.8	9.9 ± 0.7	0.52
Phosphate (mg/dl)	5.0 ± 0.9	5.0 ± 0.9	4.9 ± 0.9	0.84
Intact PTH (pg/mL)	178.4 ± 22.1	195.9 ± 237.8	91.3 ± 53.6	0.22
	121.0 (65.0, 221.0)	132.0 (76.0, 225.0)	65.0 (57.5, 112.0)	
PD vintage (months)	38.5 ± 28.1	36.6 ± 30.1	46.0 ± 17.3	0.44
D/P Cr	0.63 ± 0.14	0.64 ± 0.12	0.55 ± 0.19	0.21
ABI	1.07 ± 0.22	1.15 ± 0.13	0.72 ± 0.14	0.0001*

Values are expressed as mean ± standard deviation. Intact PTH is expressed as median and interquartile range. The values are compared between the groups by the chi-square test, t-test and Mann–Whitney U test as appropriate. *, *p*<0.05.

Abbreviations: ABI, ankle-brachial index; High-ABI group, group of patients with high ABI (ABI>0.9); Low-ABI group, group of patients with low ABI (0.9 or low); BMI, body mass index; DM, diabetes mellitus; CVD, cardiovascular and/or cerebrovascular disease; PTH, parathyroid hormone; PD, peritoneal dialysis; D/P Cr, dialysate/plasma creatinine ratio.

**Table 2:** Serum lipid levels

	**All**	**High-ABI group**	**Low-ABI group**	***p***
Total cholesterol level (mg/dl)	182.0 ± 35.5	183.2 ± 37.5	177.1 ± 27.9	0.69
CM cholesterol level (mg/dl)	0.96 ± 1.97	0.57 ± 0.48	2.54 ± 4.17	0.75
	0.42 (0.22, 1.05)	0.39 (0.22, 0.97)	0.49 (0.32, 3.81)	
VLDL cholesterol level (mg/dl)	35.51 ± 15.54	33.78 ± 14.9	42.43 ± 17.1	0.19
LDL cholesterol level (mg/dl)	92.01 ± 27.75	93.30 ± 29.0	86.84 ± 23.1	0.59
HDL cholesterol level (mg/dl)	53.46 ± 21.20	55.50 ± 22.3	45.33 ± 14.5	0.26
Triglyceride level (mg/dl)	132.39 ± 71.60	123.40 ± 40.31	168.36 ± 140.90	0.10
	118.95 (89.72, 153.11)	118.42 (94.98, 146.28)	130.06 (55.12, 256.53)	
Apolipoprotein B level (mg/dl)	88.57 ± 21.32	87.82 ± 23.30	91.57 ± 11.41	0.68
Non-HDL cholesterol level (mg/dl)	128.48 ± 36.17	127.65 ± 39.50	131.80 ± 19.39	0.79

Values are expressed as mean ± standard deviation. CM cholesterol and triglyceride levels are expressed as median and interquartile range. The values are compared between the groups by the t-test and Mann–Whitney U test as appropriate.

Abbreviations: ABI, ankle-brachial index; High-ABI group, group of patients with high ABI (ABI>0.9); Low-ABI group, group of patients with low ABI (0.9 or low); CM, chylomicron; VLDL, very-low-density lipoprotein; LDL, low-density lipoprotein; HDL, high-density lipoprotein.

### Cholesterol proportions in lipoprotein fractions and ABI

**Figure 1:**
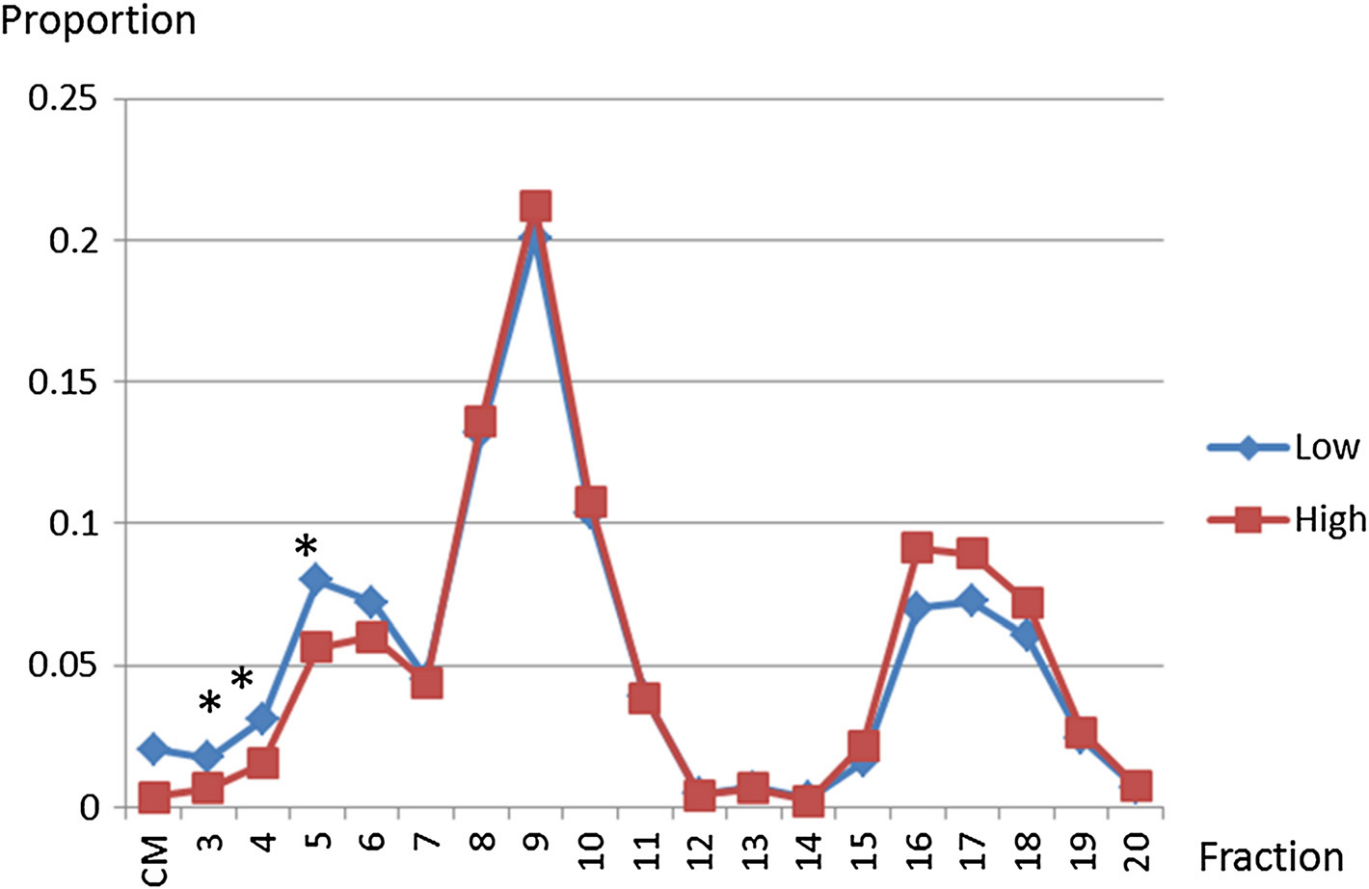
**Cholesterol proportions in different lipoprotein fractions to total cholesterol level in low-ABI group compared with those in high-ABI group.** Average proportion of cholesterol level in each lipoprotein fraction to total cholesterol level is indicated in the graph. The values are compared between the groups by the t-test and Mann–Whitney U test as appropriate. *, *p*<0.05. Abbreviations: proportion, average of the proportion of cholesterol level in each lipoprotein fraction to total cholesterol level; High-ABI group, group of patients with high ABI (ABI>0.9); Low-ABI group, group of patients with low ABI (0.9 or low); CM, chylomicron; F3-20, Fractions 3–20.

The cholesterol proportions in different lipoprotein fractions are shown in Figure 1. In large VLDLs (F3-F5), the cholesterol proportions in the low-ABI group were higher than those in the high-ABI group (F3, *p*=0.011; F4, *p*=0.038; F5, *p*=0.032). In small HDL (F18), the cholesterol proportions in the low-ABI group were marginally lower than those in the high-ABI group (*p*=0.088).

Univariate linear regression analysis showed that DM and CVD were associated with ABI (DM, parameter estimate=-0.19, *p*=0.0077; CVD, parameter estimate=-0.19, *p*=0.022). ABI was not associated with age; gender; BMI; hypertension; statin use; icodextrin use; serum creatinine, albumin, albumin-corrected serum calcium, phosphate, and intact PTH levels; and albumin-corrected serum calcium-phosphate product. Univariate linear regression analysis of lipid levels showed that ABI was negatively associated with log(CM) and VLDL-C level, and multivariate linear regression analysis adjusted for DM and CVD also showed that ABI was negatively associated with log(CM) and VLDL-C level (Table 3).

**Table 3:** Relationships between ABI and serum lipid levels

	**Univariate**		**Multivariate**	
	**Parameter estimate**	***p***	**Parameter estimate**	***p***
Total cholesterol level (mg/dl)	-0.000189 (0.00106)	0.86	-0.00104 (0.00095)	0.28
Log(CM)	-0.0701 (0.0250)	0.0084*	-0.0588 (0.0230)	0.016*
VLDL cholesterol level (mg/dl)	-0.00547 (0.00222)	0.019*	-0.00566 (0.00197)	0.0074*
LDL cholesterol level (mg/dl)	-0.000251 (0.00135)	0.85	-0.00106 (0.00118)	0.38
HDL cholesterol level (mg/dl)	0.00316 (0.00168)	0.069	0.00206 (0.00152)	0.18
Log(TG)	-0.151 (0.0770)	0.059	-0.130 (0.0679)	0.065
Apolipoprotein B level (mg/dl)	-0.00232 (0.00171)	0.18	-0.00289 (0.00147)	0.058
Non-HDL cholesterol level (mg/dl)	-0.00127 (0.00101)	0.22	-0.00167 (0.000874)	0.065

Relationships between ABI and serum lipid levels were evaluated by univariate or multivariate linear regression analysis adjusted for DM and CVD. The values are expressed as parameter estimates (standard deviation). *, *p*<0.05.

Abbreviations: Univariate, univariate linear regression analysis; multivariate, multivariate linear regression analysis; log(CM), natural logarithm value of chylomicron cholesterol level (mg/dl); VLDL, very-low-density lipoprotein; LDL, low-density lipoprotein; HDL, high-density lipoprotein; log(TG), natural logarithm value of triglyceride level (mg/dl); DM, diabetes mellitus; CVD, cardiovascular and/or cerebrovascular disease.

Univariate linear regression analysis of the cholesterol proportion in lipoprotein fractions showed that ABI was negatively associated with large VLDL (F3-F5) and medium VLDL (F6) and positively associated with medium HDL (F17), small HDL (F18), and very small HDLs (F19 and F20) (Table 4). After adjustment for DM and CVD, ABI was still associated with the cholesterol proportions in the large VLDLs, medium VLDL, and the medium HDL.

**Table 4:** Relationship between ABI and the proportion of cholesterol level in each lipoprotein fraction

			**Univariate**		**Multivariate**	
**Class**	**Subclass**	**Fraction**	**Parameter estimate**	***p***	**Parameter estimate**	***p***
CM		F1 + F2	-4.13 (2.09)	0.056	-2.05 (1.99)	0.31
VLDL	Large VLDL	F3	-8.72 (3.36)	0.014*	-5.62 (3.24)	0.92
		F4	-4.99 (1.90)	0.013*	-3.82 (1.76)	0.038*
		F5	-4.26 (1.22)	0.0014*	-3.62 (1.16)	0.0039*
	Medium VLDL	F6	-3.26 (1.43)	0.030*	-3.25 (1.24)	0.014*
	Small VLDL	F7	-3.99 (3.03)	0.20	-4.11 (2.58)	0.12
LDL	Large LDL	F8	-0.383 (1.195)	0.75	-0.554 (1.034)	0.60
	Medium LDL	F9	0.0471 (0.870)	0.96	-0.162 (0.756)	0.83
	Small LDL	F10	-0.185 (1.353)	0.89	-0.122 (1.168)	0.92
	Very small LDL	F11	-1.21 (4.07)	0.77	-1.95 (3.51)	0.58
		F12	-4.47 (11.57)	0.70	4.98 (10.36)	0.63
		F13	8.75 (30.99)	0.78	7.44 (26.75)	0.78
HDL	Very large HDL	F14	-22.23 (28.83)	0.45	-13.71 (25.22)	0.59
		F15	2.40 (1.78)	0.19	1.96 (1.54)	0.21
	Large HDL	F16	1.09 (0.59)	0.075	1.00 (0.51)	0.059
	Medium HDL	F17	3.68 (1.25)	0.0059*	3.06 (1.11)	0.0099*
	Small HDL	F18	5.36 (2.14)	0.017*	3.73 (1.97)	0.068
	Very small HDL	F19	12.48 (6.07)	0.048*	7.06 (5.72)	0.23
		F20	58.56 (25.94)	0.031*	45.23 (23.00)	0.058

Relationships between ABI and the proportion of cholesterol level in each lipoprotein fraction were evaluated by univariate or multivariate linear regression analysis adjusted for DM and CVD. The values are expressed as parameter estimates (standard deviation). *, *p*<0.05.

Abbreviations: Univariate, univariate linear regression analysis; multivariate, multivariate linear regression analysis; CM, chylomicron; VLDL, very-low-density lipoprotein; LDL, low-density lipoprotein; HDL, high-density lipoprotein; F1-20, Fraction 1–20; DM, diabetes mellitus; CVD, cardiovascular and/or cerebrovascular disease.

### Sizes of lipoprotein particles

The size of VLDL particles in the low-ABI group was larger than that in the high-ABI group (Table 5). The negative relationship between the size of VLDL particles and ABI was observed after the adjustment for DM and CVD (Table 6).

**Table 5:** Sizes of lipoprotein particles

	**All**	**High-ABI group**	**Low-ABI group**	***p***
VLDL (nm)	40.19 ± 2.09	39.83 ± 1.79	41.62 ± 2.70	0.041*
LDL (nm)	25.26 ± 0.34	25.28 ± 0.32	25.20 ± 0.43	0.58
HDL (nm)	10.85 ± 0.35	10.85 ± 0.35	10.85 ± 0.34	0.99

Values are expressed as mean ± standard deviation. The values are compared between the groups by t-test. *, *p*<0.05.

Abbreviations: ABI, ankle-brachial index; High-ABI group, group of patients with high ABI (ABI>0.9); Low-ABI group, group of patients with low ABI (0.9 or low); VLDL, very-low-density lipoprotein; LDL, low-density lipoprotein; HDL, high-density lipoprotein.

**Table 6:** Relationships between ABI and sizes of lipoprotein particles

	**Univariate**		**Multivariate**	
	**Parameter estimate**	***p***	**Parameter estimate**	***p***
VLDL (nm)	-0.0464 (0.0160)	0.0067*	-0.0352 (0.0156)	0.032*
LDL (nm)	0.0317 (0.109)	0.77	0.00181 (0.0950)	0.99
HDL (nm)	0.0642 (0.108)	0.55	0.0834 (0.0923)	0.37

Relationships between ABI and the sizes of lipoprotein particles were evaluated by univariate or multivariate linear regression analysis adjusted for DM and CVD. The values are expressed as parameter estimates (standard deviation). *, *p*<0.05.

Abbreviations: Univariate, univariate linear regression analysis; multivariate, multivariate linear regression analysis; CM, chylomicron; VLDL, very-low-density lipoprotein; LDL, low-density lipoprotein; HDL, high-density lipoprotein; DM, diabetes mellitus; CVD, cardiovascular and/or cerebrovascular disease.

The size of VLDL particles was positively associated with CM-C, VLDL-C, and TG levels, and negatively associated with HDL-C level (Table 7). The size of VLDL particles was positively associated with the cholesterol proportions in the CM, large VLDLs (F3-F5), medium VLDL (F6), and very small LDL (F12), and negatively associated with those in large LDL (F8) and large and medium HDLs (F16 and F17) (Table 8). The size of LDL particles was negatively associated with the cholesterol proportions in large VLDLs (F3 and F4), and positively associated with that in small VLDL (F7) (Table 8). The size of HDL particles was negatively associated with VLDL-C and TG levels (Table 7). The size of HDL particles was negatively associated with the cholesterol proportions in large VLDLs (F4 and F5) and medium VLDL (F6) (Table 8).

**Table 7:** Correlations between sizes of lipoprotein particles and serum cholesterol levels of lipoproteins and TG levels

	**Size of VLDL particles**		**Size of LDL particles**		**Size of HDL particles**	
	**r**	***P***	**r**	***p***	**r**	***p***
Total cholesterol level (mg/dl)	-0.0746	0.67	0.0922	0.60	-0.017	0.92
CM cholesterol level (mg/dl)	0.861	0.0001*	-0.414	0.014*	-0.351	0.039*
VLDL cholesterol level (mg/dl)	0.569	0.0004*	-0.208	0.23	-0.368	0.030*
LDL cholesterol level (mg/dl)	-0.174	0.32	0.0746	0.67	-0.354	0.037*
HDL cholesterol level (mg/dl)	-0.381	0.024*	0.259	0.13	0.734	0.0001*
Triglyceride level (mg/dl)	0.701	0.0001*	-0.365	0.031*	-0.455	0.006*

Values are expressed as Pearson’s correlation coefficient and p values. *, p<0.05.

The relationships between sizes of lipoprotein particles and CM cholesterol and triglyceride levels were evaluated by Spearman’s rank correlation coefficient.

Abbreviations: CM, chylomicron; VLDL, very-low-density lipoprotein; LDL, low-density lipoprotein; HDL, high-density lipoprotein; r, Pearson’s correlation coefficient; F3-20, Fractions 3–20.

**Table 8:** Correlations between sizes of lipoprotein particles and cholesterol proportions in lipoprotein fractions

	**Size of VLDL particles**		**Size of LDL particles**		**Size of HDL particles**	
	**r**	***p***	**R**	***P***	**r**	***p***
CM	0.674	0.0001*	-0.534	0.001*	-0.310	0.070
F3	0.840	0.0001*	-0.573	0.0003*	-0.333	0.050
F4	0.878	0.0001*	-0.563	0.0004*	-0.348	0.041*
F5	0.795	0.0001*	-0.277	0.11	-0.392	0.020*
F6	0.462	0.0052*	-0.238	0.17	-0.398	0.018*
F7	-0.167	0.34	0.491	0.0028*	-0.0650	0.71
F8	-0.584	0.0002*	0.757	0.0001*	0.0707	0.69
F9	-0.277	0.11	0.762	0.66	-0.457	0.0057*
F10	0.234	0.18	-0.610	0.0001*	-0.793	0.0001*
F11	0.270	0.12	-0.602	0.0001*	-0.799	0.0001*
F12	0.382	0.023*	-0.648	0.0001*	-0.473	0.0041*
F13	0.0473	0.79	-0.266	0.12	0.0679	0.70
F14	0.160	0.36	-0.134	0.44	0.177	0.31
F15	-0.290	0.092	0.310	0.070	0.826	0.0001*
F16	-0.419	0.012*	0.427	0.011*	0.911	0.0001*
F17	-0.433	0.0094*	0.183	0.29	0.575	0.0003*
F18	-0.0527	0.76	-0.408	0.015*	-0.220	0.21
F19	-0.0149	0.93	-0.313	0.067	-0.239	0.17
F20	-0.0404	0.82	-0.255	0.14	0.129	0.46

Values are expressed as Pearson’s correlation coefficient and *p* values. *, *p*<0.05.

The relationships between sizes of lipoprotein particles and cholesterol proportions in CM were evaluated by Spearman’s rank correlation coefficient.

## Discussion

In this study, we identified the relationships between ABI and lipid profiles in PD patients by HPGPC. There has been no detailed report of lipid profiles in PD patients with controlled serum LDL-C levels. ABI was negatively associated with VLDL-C level. We were able to determine that the cholesterol proportions in VLDLs were higher in the low-ABI group than in the high-ABI group, and that ABI was negatively associated with VLDL-C level, cholesterol proportions in the large and medium VLDLs and the size of VLDL particles. Serum TG level was positively associated with the sizes of VLDL particles and negatively associated with those of LDL and HDL. The relationship between the size of VLDL particles and the cholesterol proportion in the very small LDL fraction was observed. The size of LDL particles was negatively associated with the cholesterol proportion in the large VLDLs, and the size of HDL particles was negatively associated with the cholesterol proportion in the large and medium VLDLs.

CM transports dietary TG from the gut to the liver and peripheral tissue in the exogenous pathway of lipoprotein metabolism. VLDL is produced by the liver and serves as a vehicle for the delivery of endogenous lipids to the peripheral tissue in the endogenous pathway of lipoprotein metabolism. In this study, we found that the patients in the low-ABI group tended to show high cholesterol proportions in the VLDLs, and that cholesterol proportions in the VLDLs were associated with ABI in PD patients. It has been reported that, in PD patients, glucose absorption from dialysate stimulates hepatic lipoprotein and TG synthesis [20,21]. These results suggest that glucose absorption from dialysate activates the endogenous pathway of lipoprotein metabolism in PD patients, and that the exogenous pathway of lipoprotein metabolism may aggravate atherosclerosis though the endogenous pathway.

There have been two reported routes through which VLDL contributes to the progression of atherosclerosis, namely, through intermediate density lipoprotein (IDL) and small dense LDL [18,22]. The lipolysis and remodeling of TG-rich VLDL produce IDL and LDL. Concerning the IDL route to atherosclerosis, it has been reported that human atherosclerosis plaques contain TG-rich lipoproteins [17]. In rabbits, TG-rich lipoprotein remnants can penetrate the arterial wall [19]. However, in this study, we were unable to evaluate the relationship between IDL level and ABI because HPGPC does not enable the direct measurement of IDL.

Under hypertriglyceridemia, the large TG-rich VLDL1 subfraction is mainly produced. Then, VLDL1 generates the slowly metabolized, small dense LDL subspecies; however, large and light LDLs, which are rapidly cleared from the plasma, appear to result from the delipidation of either IDL or VLDL2 [23]. In this study, the relationships between TG level and the sizes of VLDL and LDL particles were observed. The results of this study were consistent with previous results and suggested that the size of VLDL particles increases with an increase in serum TG level, and that the increase in large VLDL level may induce small LDL particles.

It has been reported that the hepatic production of VLDL1 is related to insulin resistance [24]. VLDL2 is not related to insulin resistance. In PD patients, a high glucose load absorbed from dialysate causes metabolic abnormalities. It has been reported that PD patients have a higher homeostasis model assessment index than HD patients [25]. It has also been reported that, after PD initiation, new-onset hypertriglyceridemia is observed among PD patients [26,27]. These lines of evidence suggest that, in PD patients, glucose absorption from dialysate induces hypertriglyceridemia and insulin resistance followed by an increase in VLDL1 and small dense LDL levels.

In this study, no relationships between the cholesterol proportions in the LDL fractions and ABI were observed. One of the reasons may be that most of the patients with dyslipidemia were treated with a statin in this study. It has been reported that statins decrease total LDL level [28]. The effect of statins on LDL may have masked the effect of small dense LDL on atherosclerosis.

The size of HDL particles has been reported to be affected by serum TG level [29]. An increased serum TG level leads to a decrease in large HDL2 level and an increase in small HDL3 level. Cholesterol ester transfer protein transfers TG from TG-rich lipoproteins to HDL [30]. Then, TG-enriched HDL is lipolyzed by hepatic lipase. In this study, the size of HDL particles was negatively associated with serum TG level and the cholesterol proportions in the VLDLs. These results suggest that a high serum TG level affects HDL metabolism in PD patients.

The results of this study showed that, among PD patients with controlled LDL-C levels, VLDL was associated with atherosclerosis, and that a high serum TG level atherogenically affected the size of lipoprotein particles. LDL-C level has been used for the statin treatment goal of dyslipidemia in ESRD patients. However, it has been reported that statin treatment with lowering LDL-C level has little or no effect on all-cause mortality, cardiovascular mortality, or cardiovascular events in individuals receiving dialysis [31]. Additional markers to LDL-C level and additional therapy with statins are needed to treat dyslipidemia in ESRD patients. This study suggested that VLDL-C level may be a useful candidate marker of atherosclerosis to treat dyslipidemia in ESRD patients.

It has been reported that PD patients with a high TG level have a high risk of death [32]. A cohort study of PD patients showed that ABI is associated with serum TG level [6]. Therefore, lowering serum TG level can be a target against atherosclerosis in PD patients after the control of LDL-C level. Fibrate is often used to lower serum TG level. Fenofibrate has been reported to reduce serum TG level by 38% and small dense LDL-C level by 23% without changing LDL-C level, and to increase serum HDL-C level by 14% in non-dialysis patients with type 2 DM [28]. However, care should be taken to prevent the development of adverse effects of fibrates such as rhabdomyolysis in PD patients treated with statins. An observational study of PD patients with DM showed that serum LDL-C and TG levels decrease following an overnight dwell with icodextrin [33]. A randomized controlled trial showed a decreased serum TG level in diabetic PD patients treated with icodextrin [34]. The inhibition of glucose absorption may be effective in lowering serum TG level. More studies are required to evaluate the effects of lowering serum TG level on the prevention of PAD and atherosclerosis.

This study has several limitations. First, as with any cross-sectional study, we were unable to examine the longitudinal changes in laboratory findings over time. Second, this study involved 35 patients, which may be insufficient for detecting the relationship between ABI and lipoprotein fraction. Third, geographical and selection bias may have been included in this study. Fourth, we did not investigate the caloric intake such as diet or glucose load of dialysate. We were also unable to adjust for the effects of caloric intake on dyslipidemia. Fifth, we did not investigate insulin resistance and thus were unable to evaluate the effects of insulin resistance on lipoprotein fraction. Sixth, because the DM patients were only 15 in this study, the effect of antihyperglycemic drugs on dyslipidemia was not investigated. Seventh, most of the patients in this study had anuria. We were unable to evaluate the effect of residual renal function on lipid metabolism. Eighth, hypothyroidism affects dyslipidemia in ESRD patients. However, we were unable to carry out thyroid function tests.

## Conclusions

This study showed that, among PD patients with controlled LDL-C levels, VLDL was associated with atherosclerosis, and that a high serum TG level had an atherogenic effect on the size of lipoprotein particles. Because PD patients tend to have high serum TG levels caused by glucose absorption from dialysate, their endogenous pathway of lipoprotein metabolism may be activated to produce VLDL, which may lead to the progression of atherosclerosis. Lowering serum TG level may be an effective therapy against atherosclerosis in PD patients after the control of LDL-C level.

## Abbreviations

PAD: Peripheral artery disease; PD: Peritoneal dialysis; ABI: Ankle-brachial index; LDL: Low-density lipoprotein; Cholesterol Proportion: Proportions of cholesterol levels to total cholesterol level; VLDL: Very-low-density lipoprotein; ESRD: End-stage renal disease; TASC2: Trans-Atlantic Inter-Society Consensus 2 for the management of PAD; DM: Diabetes mellitus; TG: Triglyceride; LDL-C: LDL cholesterol; HDL-C: High-density lipoprotein cholesterol; K/DOQI: Kidney disease outcomes quality initiative; HPGPC: High-performance gel permeation chromatography; HDL: High-density lipoprotein cholesterol; CVD: Cardiovascular and/or cerebrovascular diseases; PTH: Parathyroid hormone; CM: Chylomicron; F1: Fraction 1; VLDL-C: VLDL cholesterol; CM-C: CM cholesterol; log(CM): natural logarithm value of CM-C level; log(TG): natural logarithm value of TG level; High-ABI group: a group of patients with high ABI (ABI>0.9); Low-ABI group: a group of patients with low ABI (0.9 or low); BMI: Body mass index; D/P Cr: dialysate/plasma creatinine ratio; univariate: univariate linear regression analysis; multivariate: linear regression analysis; r: Pearson’s correlation coefficient.

## Competing interests

No financial or other interests to be declared.

## Authors’ contributions

Each author contributed to this manuscript. EK and YM collected the data. EK wrote this manuscript. EK, MA and MO contributed to the statistical analysis and interpretation of the data. YM, SS and MY contributed to the conception and design of the study and revised this study. All authors reviewed and approved the manuscript.

## Pre-publication history

The pre-publication history for this paper can be accessed here:

http://www.biomedcentral.com/1471-2369/14/212/prepub
